# The Use of a DNA-Intercalating Dye for Quantitative Detection of Viable *Arcobacter* spp. Cells (v-qPCR) in Shellfish

**DOI:** 10.3389/fmicb.2019.00368

**Published:** 2019-02-28

**Authors:** Nuria Salas-Massó, Quyen Than Linh, Wai Hoe Chin, Anders Wolff, Karl B. Andree, M. Dolors Furones, María José Figueras, Dang Duong Bang

**Affiliations:** ^1^Unitat de Microbiologia, Departament de Ciènces Médiques Bàsiques, Facultat de Medicina i Ciències de la Salut, Institut d’Investigació Sanitària Pere Virgili, Universitat Rovira i Virgili, Reus, Spain; ^2^IRTA-Sant Carles de la Ràpita, Sant Carles de la Ràpita, Spain; ^3^Department of Bioengineering and Biomedicine, Technical University of Denmark, Lyngby, Denmark; ^4^Zoetis Denmark Gammelgårdsvej, Farum, Denmark; ^5^Division of Microbiology, National Food Institute, Technical University of Denmark, Lyngby, Denmark

**Keywords:** PMA, qPCR, viable cells, *Arcobacter*, shellfish

## Abstract

The genus *Arcobacter* (Vandamme et al., 1991), comprised of *Campylobacter*-related species, are considered zoonotic emergent pathogens. The presence of *Arcobacter* in food products like shellfish, has an elevated incidence worldwide. In this study, we developed a specific viable quantitative PCR (v-qPCR), using the dye propidium monoazide (PMA), for quantification of the viable *Arcobacter* spp. cells in raw oysters and mussels. The high selectivity of primers was demonstrated by using purified DNA from 38 different species, 20 of them from the genus *Arcobacter*. The optimization of PMA concentration showed that 20 μM was considered as an optimal concentration that inhibits the signal from dead cells at different concentrations (OD_550_ from 0.2 to 0.8) and at different ratios of live: dead cells (50:50 and 90:10). The v-qPCR results from shellfish samples were compared with those obtained in parallel using several culture isolation approaches (i.e., direct plating on marine and blood agar and by post-enrichment culturing in both media). The enrichment was performed in parallel in Arcobacter-CAT broth with and without adding NaCl. Additionally, the v-qPCR results were compared to those obtained with traditional quantitative (qPCR). The v-qPCR and the qPCR resulted in c.a. 94% of positive detection of *Arcobacter* vs. 41% obtained by culture approaches. When examining the reduction effect resulting from the use of v-qPCR, samples pre-enriched in Arcobacter-CAT broth supplemented with 2.5% NaCl showed a higher reduction (3.27 log copies) than that of samples obtained directly and those pre-enriched in Arcobacter-CAT broth isolation (1.05 and 1.04). When the v-qPCR was applied to detect arcobacter from real shellfish samples, 15/17 samples tested positive for viable *Arcobacter* with 3.41 to 8.70 log copies 1g^-1^. This study offers a new tool for *Arcobacter* surveillance in seafood.

## Introduction

Foodborne disease outbreaks are of public health concern (Zeng et al., 2016 and references therein). In 2015, a total of 4,362 food-borne disease outbreaks, including waterborne disease outbreaks were reported in the European Union (EU). Overall, these outbreaks caused 45,874 cases of illness, 3,892 hospitalizations and 17 deaths (EFSA and ECDC, 2016). Most of the outbreaks reported in 2015 were caused by bacterial agents (33.7% of all outbreaks). The most frequent human foodborne illnesses in order of prevalence were campylobacteriosis, salmonellosis, yersiniosis, shiga toxin-producing *Escherichia coli* infections and listeriosis (EFSA and ECDC, 2016). To avoid the occurrence of disease outbreaks, food is monitored following specific microbiological criteria, which may vary according to culture, climate and economic status of the country (Zhang et al., 2016). In these regulated monitoring programs, the most commonly used methods are based on bacterial isolation in synthetic media, which are time consuming, laborious and cannot detect viable-but-non-culturable bacteria (VBNC) (Barbau-Piednoir et al., 2014).

Molecular methods have been progressively introduced as they are fast, sensitive and specific. Among such methods, PCR is the most widely used. By using PCR, the presence of a pathogen of interest in a sample can be detected rapidly. However, the method is not able to give us a clear picture of the status of the bacterial population, since the method amplifies the DNA from both living and dead cells (Nocker et al., 2006; Fittipaldi et al., 2012; Elizaquível et al., 2014; Zhang et al., 2016; Reyneke et al., 2017). In a food safety context, it is important to know whether the bacteria are still alive in the food, to avoid unnecessary product recalls and economic losses (Zhang et al., 2016). Therefore, RNA-based methodologies are recommended for detecting and determining the number of viable bacterial cells that are metabolically active in the sample. The problem of RNA-based methodologies is that the RNA molecules are easily degraded, and the RNA degradation can easily occur while handling the samples (Fittipaldi et al., 2012; Barbau-Piednoir et al., 2014).

Nocker et al. (2006) were the first to use propidium monoazide (PMA) for examining the suitability of this membrane-impermeant dye to intercalate to genomic DNA from cells with compromised cell membranes as an alternative tool to discriminate between viable and dead cells. The basic ideas of the use of this dye are that (i) the dye is able to covalently bind to DNA after photoactivation using light with 450 nm wavelength, and (ii) the dye is usually not permeable to intact cell membranes, so the dye only can enter into membrane-compromised cells (i.e., dead or damaged cells). Once the PMA is inside the cells it intercalates into the DNA and, after photoactivation it is crosslinked to the DNA. This chemical modification will block (inhibit) the amplification of these DNA molecules during PCR. At the same time crosslinking occurs, the remaining PMA in the solution reacts with water and becomes unreactive (Nocker et al., 2009).

*Arcobacter* is a new foodborne pathogen. It is related to *Campylobacter* that is one of the main causes of diarrhea in humans. *Arcobacter butzleri* has been the cause of enteritis outbreaks associated to the consumption of contaminated water and food in different countries (Collado and Figueras, 2011; Ferreira et al., 2016). Recently Ferreira et al. (2017) reported how *Arcobacter* is commonly isolated along the whole food production chain, including animals from farms, slaughterhouses and retail. Although *Arcobacter* spp. have been isolated from poultry, pork, dairy products, and vegetables (Collado et al., 2009; Wesley and Miller, 2010; Hsu and Lee, 2015; Ferreira et al., 2017), their prevalence rate in seafood products, some of which are consumed raw or undercooked, is relatively high compared to other foods, ranging from 14.6 to a 73.3% of positive samples (Collado et al., 2009; Nieva-Echevarria et al., 2013; Salas-Massó et al., 2016, 2018; Leoni et al., 2017). In these types of food samples *A. butzleri* has been shown to be the most prevalent species using conventional culture methods, however, other species may prevail using other approaches (Salas-Massó et al., 2016). So far, there are no official standard protocols for the isolation of these bacteria. Some of the developed methods are time consuming, as they require at least 48 h for growing cultures and a pre-enrichment step in a broth containing antibiotics (Levican et al., 2016; Salas-Massó et al., 2016). Therefore, advances in molecular tools for the study of these bacteria have been developed (Ferreira et al., 2017). So far, few publications report the use of DNA-intercalating dyes to study the viability of *Arcobacter* spp. cells. Hrušková et al. (2013) used a PMA methodology for detection of *A. butzleri* and *A. cryaerophilus* in biofilms and studied their composition in relation to the viability status of the cells. Recently, Webb et al. (2016) used ethidium monoazide (EMA) coupled with a qPCR to evaluate how wastewater treatments can affect the viability of *A. butzleri* cells. Despite the high prevalence of *Arcobacter* spp. in seafood, there are no studies that have used PMA to investigate these bacteria in shellfish. In fact, up to date only two PMA treatments have been developed for the study of other microbes in seafood samples. Zhu et al. (2012) assayed a PMA-qPCR in raw oysters to quantify viable cells of *Vibrio parahaemolyticus* positive for the thermostable direct hemolysin gene (*tdh*) which is associated to the pathogenicity of this organism. Quijada et al. (2016) developed a PMA-qPCR to detect and enumerate enteric RNA and DNA viruses in clams, both strategies showing promising results as alternatives to predicting the status of viability of these foodborne pathogens.

The aim of the study is to define the best conditions under which the PMA method can be used and to develop a PMA-qPCR protocol for the detection and enumeration of viable *Arcobacter* cells in seafood samples, comparing the results with those obtained with different culture approaches.

## Materials and Methods

### Bacterial Strains and Culture Conditions

A total of 38 bacterial species, 18 species comprising reference strains from eight different genera, like *Campylobacter* related to *Arcobacter* and others frequently recovered from shellfish (i.e., *Salmonella, Escherichia coli*, etc.), and 20 *Arcobacter* species. The bacterial strains were used to develop and examine the specificity of primers and probes (Supplementary Table S1). The different strains were grown on tryptone soy agar at 37°C for 24 h (TSA, Difco, France), with the exception of *Arcobacter* species that were grown on Blood Agar (BA; TSA supplemented with 5% sheep blood BD Difco, Le Pont de Claix, France), and *A. marinus* and *A. halophilus*, that were grown on Marine Agar (MA; Scharlab, Barcelona, Spain) and were incubated under aerobic conditions at 30°C for 48 h. Species from the genus *Campylobacter* were inoculated on BA and incubated under microaerobic conditions (oxygen, 6 to 16%; carbon dioxide, 2 to 10%; and nitrogen, 80%; generated using the Gas Pak EZ Campy container sachets^TM^ Becton Dickinson, Sparks, MD, United States) at 37°C for 48 h.

### Sample Preparation and Analysis

#### Pure Culture Samples and Mixed Models

**FIGURE 1:**
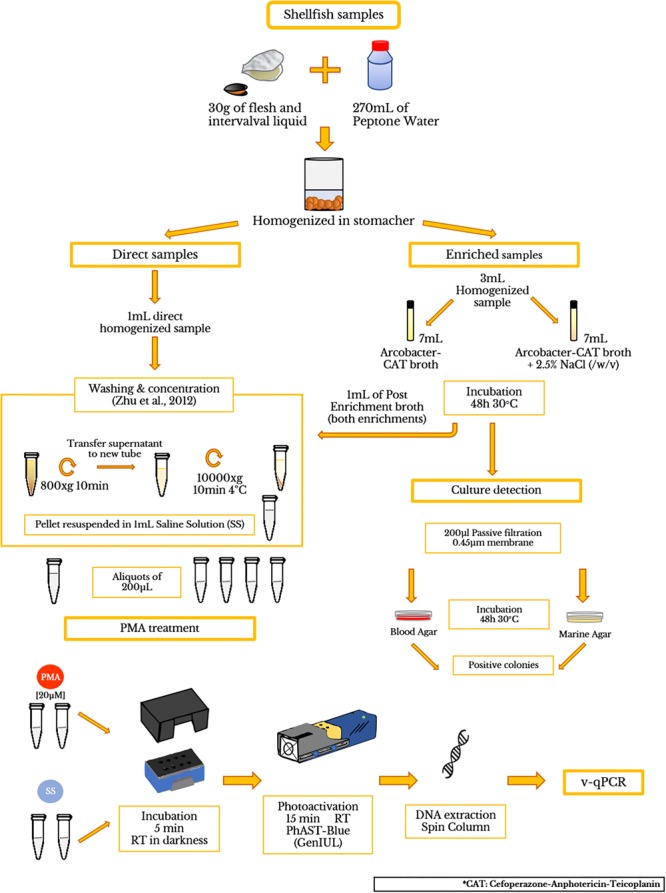
Flow diagram showing the processing of shellfish samples and PMA treatment.

Twenty *Arcobacter* species (Supplementary Table S1) were used to determine the viability of cells, and evaluate the specificity of the developed assay. Initial bacterial suspensions were prepared in 0.9% (w/v) sterile saline solution (SS) up to an OD_550_ = 0.250. An aliquot of those live cell (LC) suspensions was used to obtain dead cells (DC) by thermal inactivation (100°C, 10 min). Four models, 100% LC; 50% LC + 50% DC; 10% LC + 90% DC and 100% DC in a final volume of 200 μl were tested for each species in duplicate. For quantification of CFU and confirm the efficacy of thermal inactivation, the LC and DC suspensions were plated on BA (with the exception of *A. marinus* and *A. halophilus* that were plated on MA) and incubated at 30°C for 48 h, (Figure 1). The procedure was continued as described in the sections “PMA treatment” and “DNA isolation.” Additionally, LC treated with PMA were also plated on the media described above to check for any cytotoxic effects on the cells by the presence of this DNA-intercalating dye.

#### Artificially Contaminated Samples

Depurated oyster and mussel samples were collected from a depuration plant in Alfacs Bay (Ebro River Delta, Spain) and were scrubbed, shucked, and then homogenized with a stomacher (Lab ⋅ Blender 400). A mix of 270 mL of peptone water (PW) and 30 g of flesh and intervalval liquid from the seafood were homogenized (ISO/TS 16649-3:2005). Then, the homogenized mixtures (hereinafter referred to as direct samples) were aliquoted in 9 mL and were inoculated with 1 mL of a 10-fold dilution (from 10^0^ to 10^-6^) from an initial inoculum (1.26 × 10^8^ CFU/ml) of *A. butzleri* (OD_550_ = 0.250). Additionally, a mix of living and dead *A. butzleri* cells (100% LC; 50% LC + 50% DC; 10% LC + 90% DC; 100% DC) were also inoculated in the equivalent homogenized mixtures of shellfish. The CFU number in each dilution was obtained by standard plate counting methods using BA plates, which were incubated for 48 h at 30°C. After seeding and agitating, bacterial cells were obtained following the washing and concentration protocol, previously described by Zhu et al. (2012). To test the presence of background *Arcobacter* spp. in the depurated samples, 3 mL aliquots of homogenized sample were transferred to 7 mL of Arcobacter-CAT broth and Arcobacter-CAT broth supplemented with 2.5% NaCl and incubated at 30°C for 48 h to collect cell pellets for qPCR testing (hereinafter referred to as blank samples). Additionally, 200 μL of the enrichment broth were plated on BA and MA for examining positive culture of *Arcobacter* spp. The final pellets were suspended in 1 mL of SS, for further analysis (see sections “PMA Optimization Protocol,” “DNA Isolation,” “Polymerase Chain Reaction (PCR),” “Quantitative PCR (qPCR)”) A flow diagram is shown in Figure 1.

#### Natural Samples

To study the effectiveness of PMA-qPCR, 17 different raw seafood samples including oyster (*Crassostrea gigas*; *n* = 2), mussel (*Mytilus galloprovincialis*; *n* = 5), razor clam (*Ensis arcuatus*; *n* = 3) and wedge clam (*Donax trunculus*; *n* = 7) collected from Alfacs Bay were tested. Analysis was conducted within 24 h after the collection. The samples were scrubbed, shucked, and homogenized as mentioned above in Section “Artificially Contaminated Samples.” After the washing treatment, the pellets were suspended in 1 mL of sterile salt water. The presence of background *Arcobacter* was performed for all the samples as mentioned in Section “Artificially Contaminated Samples.”

### PMA Treatment

PMA (Blu-V Viability PMA Kit, Qiagen, Germany) was suspended in 20% dimethyl sulfoxide (DMSO) according to the manufacturer’s instructions to a final concentration of 2 mM, which was used as stock and was stored at -20°C in the dark. The PMA solution was added to 200 μL of sample in a 1.5 mL light-transparent micro-centrifuge tube to yield a final concentration of 20 μM. The tubes were incubated at room temperature in the dark for 5 min to allow PMA penetration into the damaged cells. Afterward, the samples were photoactivated using a PhAST-Blue lamp (450λ LED, GenIUL, Spain) for 15 min at room temperature. Duplicates of all these samples without the addition of PMA followed the same protocol.

### PMA Optimization Protocol

For the optimization of the method, conventional PCR was used as a standard [see section “Polymerase Chain Reaction (PCR)”]. The PMA was added to a final volume of 50 μL of a 1 ng/μL solution of *A. butzleri* DNA, as previously described (Nocker et al., 2006) to obtain a series of final concentrations of the dye of: 0.02, 0.2, 2, and 20 μM. An incubation time of 5 min at room temperature and a photoactivation period of 15 min, were initially applied, following the manufacturer’s instructions. Afterward, different DNA concentrations, ranging from: 1, 2, 10, and 20 ng/μL, were tested with PMA added to a final concentration of: 0.2, 2, and 20 μM, in a final volume of 50 μL.

To check whether the exposure time of the dye to light can influence the removal of the DNA signal at lower PMA concentrations, an experimental design was prepared. A series of tubes with a final volume of 50 μL containing 10 ng/μL of DNA and PMA to a final concentration of 0.2 μM were prepared. Photoactivation was performed during 7.5, 15, 30, and 60 min. Additionally, the reactivity of possible remaining excess PMA was assessed according to Nocker et al. (2006). Briefly, tubes containing 47.5 μL of PMA at 0.02, 0.2, 2, and 20 μM concentration were photoactivated for 15 min. Afterward, 2.5 μL of 20 ng/μL of DNA was added and photoactivated again, to see if there was remaining cross-linking activity of PMA to DNA. All experiments described included a positive and negative control and were performed twice.

### DNA Isolation

Total bacterial DNA was extracted from natural and artificially spiked samples (including direct samples and cell pellets from the enrichment in Arcobacter-CAT and Arcobacter-CAT supplemented with 2.5% NaCl; sections “Artificially Contaminated Samples” and “Natural Samples”) with the isolation performed according to Zhu et al. (2012) using spin columns (QIAmp DNA MiniKit 250; Qiagen, Germany) and following the manufacture’s instruction. DNA concentration was determined using a NanoDrop^TM^ 2000 spectrophotometer (Thermo Scientific, Waltham, MA, United States).

### Polymerase Chain Reaction (PCR)

Conventional PCR targeting 23S rRNA was performed to check specificity and to optimize the PMA protocol. The primers previously described by Hausdorf et al. (2013) with a modification (underlined) on the forward primer (23SF 5′-AACATATAAGCGCGATGTGGGGAC-3′; and the reverse primer: 23SR 5′-ACGGTACGGGCAACATATAATA-3′) were used. The PCR was performed in a T3 Thermocycle Biometra with PCR reaction mixtures containing 5 μL of 2X Phusion^®^ Human Specimen PCR Buffer (Thermo Fisher Scientific) forward and reverse primers to a final concentration of 500 nM, 0.1 U of Phusion Hot Start II High Fidelity DNA polymerase (Thermo Fisher Scientific), 2 μL of the DNA or DNA-PMA mix, and water to a final volume of 10 μL. The PCR program of 24 cycles consisted of: (1) 5 min at 98°C, (2) 30 s at 98°C, (3) 20 s at 56°C, (4) 20 s at 72°C, without a final elongation step. The expected PCR amplicons of 233 bp were visualized in a 2.5% agarose gel stained with RedSafe^TM^ (iNtRON Biotechnology, South Korea).

### Quantitative PCR (qPCR)

Quantitative PCR amplifications were carried out in duplicate on a CFX96 Touch Real-Time PCR Detection System (Bio-Rad, Alcobendas, Spain) in a final volume of 20 μL containing 2 μL of DNA or DNA-PMA mix, 900 nM of the modified 23S rRNA primer pair described above, 10 μL of SsoAdvanced^TM^ Universal SYBR Green Supermix (Bio-Rad), and 1 U platinum Taq DNA polymerase (Invitrogen). All assays also systematically included a negative control. The PCR thermocycling was initiated at 98°C for 3 min, followed by 35 cycles of 15 s at 95°C, 30 s at 56°C, and 1 min at 60°C. Fluorescence data were collected at the end of each cycle. The limits of detection (LOD) and quantification (LOQ) were calculated as described before (Hausdorf et al., 2013). To evaluate the effect of PMA treatment on a sample, the ΔCt was calculated. The ΔCt of a sample is the difference between the Ct-value obtained with PMA treated sample and the Ct-value obtained with non-treated sample: ΔCt = (Ct sample w/PMA – Ct sample w/o PMA). An increase in Ct values is perceived as a reduction in PCR signal and will be described as such throughout the text.

### Statistics Analysis

Ct values are presented as mean and standard deviations. Equalities of variance for the mean percentages for the model mixture experiments were assessed using the Levene’s test. Statistically significant difference in group means was performed using a one-way ANOVA analysis. To evaluate where the differences occurred between species the *post hoc* test Games-Howell was run. All the analyses were performed using Software SPSS Statistical (IBM Analytics).

## Results

### Development and Optimization of Conditions for Use of PMA to Study DNA of *Arcobacter* spp. in the Samples

It was necessary to develop and optimize a method to use the PMA for studying live and dead *Arcobacter* spp. cells. The developed method was first applied to pure DNA samples and afterward was used to discriminate live and dead cells. Initially, the minimum PMA concentration that can effectively remove the signals from 1 ng/μL of *A. butzleri* DNA was 0.2 μM (Supplementary Figure S1A). However, in the experiment testing different DNA concentrations, total removal of signal from the sample containing 20 ng/μL of *A. butzleri* DNA was achieved when using 20 μM PMA in a 50 μl volume reaction (Supplementary Figures S1B,C).

When the different photo-activation times were assessed, it was observed that periods longer than 15 min did not improve the removal of the DNA signal by PMA (Supplementary Figure S1D). As shown in Supplementary Figure S1E, after photoactivation any potential remaining excess PMA had reacted with water and was no longer effective.

### Optimization of PMA Conditions for *Arcobacter* spp. Pure Cultures

The optimized conditions described for the DNA in Section “PMA Optimization Protocol” were applied to pure *A. butzleri* cultures. Initially, cells of *A. butzleri* were resuspended in SS solution (0.9%) to a final OD_550_ = 0.250 (McFarland 1). Two different samples consisting of live cells (LC) and dead cells (DC), the latter being obtained by heating at 100°C for 10 min as described previously (Hrušková et al., 2013), were treated with PMA in a final volume of 500 μL. The PMA prevented amplification of a signal from the DNA of the dead cells as shown in Supplementary Figure S2A. Afterward, the PMA protocol was also tested at higher concentrations of dead *A. butzleri* cells (OD_550_ = 0.8, 0.4, 0.250; Supplementary Figure S2B), and resulted in the inhibition of these higher concentrations. The same results were obtained for the other 19 *Arcobacter* spp. (data not shown). After plating both LC and LC + PMA, no cytotoxic effect of the 20 μM PMA was observed, as the CFU values were equal in both cases (data not shown).

### Specificity and Viability of *Arcobacter* Cells in Model Mixtures by v-qPCR

**FIGURE 2:**
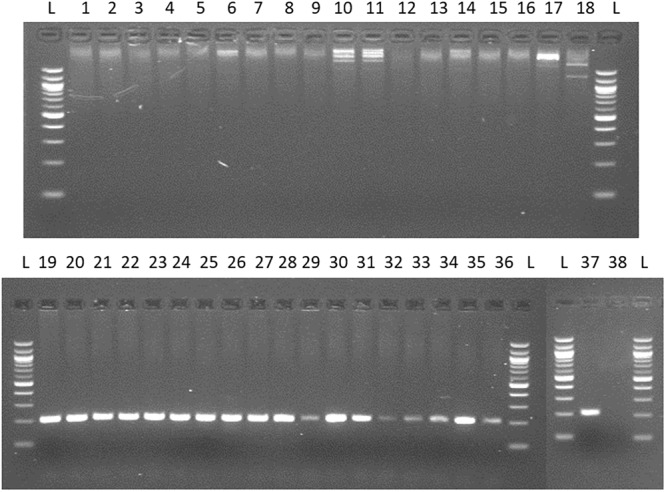
Testing specificity of 23S primers with species from genus frequently recovered/related to *Arcobacter* and 20 species of the genus *Arcobacter*. Lane 1: *Campylobacter coli;* 2: *C. jejuni;* 3: *C. lari;* 4: *C. mucosalis;* 5: *C. upsaliensis*; 6: *C. sputorum ss.spo;* 7: *C. fetus subsq. Fetus*; 8: *C. concisus;* 9: *C. hyointestinalis;* 10: *Salmonella enteritidis;* 11: *S. typhimurium;* 12: *Enterococcus faecalis;* 13: *E. faecium;* 14: *Escherichia coli;* 15: *Streptococcus pneumoniae;* 16: *Proteus hauseri;* 17: *Citrobacter freundii;* 18: *Yersinia ruckeri*. 19. *A. butzleri*; 20. *A. skirrowii*; 21 *A. cryaerophilus;* 22: *A. nitrofigilis;* 23: *A. thereius*; 24: *A. cloacae*; 25: *A. trophiarum*; 26: *A. cibarius*; 27: *A. suis*; 28: *A. defluvii*; 29: *A. marinus*; 30: *A. aquamarinus*; 31: *A. halophilus*; 32: *A. ebronensis*; 33: *A. molluscorum*; 34: *A. venerupis*; 35: *A. ellisii*; 36: *A. mytili*; 37: *A. bivalviorum*; 38: negative control.

For the quantification of viable *Arcobacter* spp. cells in seafood, a modified 23S q-PCR coupled with PMA was used. As shown in Figure 2, other Gram-negative bacteria genera, different than *Arcobacter* gave no amplification, showing the specificity of the primers. The efficiency, LOD and LOQ of the v-qPCR are shown in Table 1.

**Table 1:** Specificity, efficiency and limit of detection of Arcobacter-23S v-qPCR assays developed in this study.

23S v-qPCR
*R*^2^	0.9764
Intercept	39.72
Slope	–3.5139
PCR efficiency (%)	100.03
Limit of detection (LOD; n° copies)	21
Limit of quantification (LOQ; n° copies)	458

*Assays were tested on genomic DNA from the selected bacterial strains.*

Table 2 shows an average Ct value for *Arcobacter* species of 14.36, which ranged from 12.54 for *A. cryaerophilus*, to 17.99 for *A. nitrofigilis*. When the v-qPCR was used in model mixtures, it was observed that PMA was able to reduce the signal from dead cells, in all the different ratios that were tested (Table 2). However, the model mixture 50% LC + 50% DC was the one which gave a higher standard deviation, wherein *A. suis* there was detected a percentage of copies that was statistically (*P* < 0.05) higher than the expected for the 50% LC (71.31%). On the other hand, *A. ellisii* and *A. molluscorum* showed a lower percentage of copies detected than the expected (30.02 and 24.99%, respectively; Table 2).

**Table 2:** Bacterial model mixtures for determining the efficiency of the Arcobacter-23S v-qPCR to detect DNA from live cells.

	Ct-value	100% LC
Species	OD_550_ = 0.250	copy numbers	50% LC ^a^	10% LC ^a^	100% DC ^a^
*A. aquimarinus*	14.23 ± 0.13	2.24 × 10^7^	56.81 ± 22.45	15.43 ± 4.34	0.01 ± 0.01
*A. bivalviorum*	14.77 ± 0.18	1.41 × 10^7^	66.04 ± 16.90	15.76 ± 5.26	0.01 ± 0.01
*A. butzleri*	13.33 ± 0.07	1.17 × 10^7^	45.28 ± 5.54	11.85 ± 4.75	0.02 ± 0.01
*A. cibarius*	13.98 ± 0.32	3.11 × 10^7^	59.11 ± 5.24	11.26 ± 1.56	0.04 ± 0.03
*A. cloacae*	13.46 ± 0.28	4.21 × 10^7^	61.82 ± 11.73	14.58 ± 4.48	ND
*A. cryaerophilus*	12.54 ± 0.16	2.14 × 10^7^	51.48 ± 4.54	8.39 ± 1.83	0.01 ± 0
*A. defluvii*	13.62 ± 0.21	3.96 × 10^7^	40.08 ± 6.69	8.74 ± 1.01	0.24 ± 0.21
*A. ebronensis*	14.34 ± 0.21	1.88 × 10^7^	38.56 ± 8.09	7.11 ± 1.06	ND
*A. ellisii*	12.83 ± 1.08	7.53 × 10^7^	30.02^∗^ ± 4.53	8.16 ± 2.49	0.02 ± 0.01
*A. halophilus*	14.01 ± 0.14	2.10 × 10^7^	67.42 ± 4.84	12.61 ± 1.17	ND
*A. lanthieri*	13.54 ± 0.12	4.97 × 10^7^	47.85 ± 1.69	8.16 ± 0.62	0.01 ± 0
*A. marinus*	15.98 ± 0.13	5.62 × 10^6^	71.43 ± 16.52	23.66 ± 1.96	0.5 ± 0.27
*A. molluscorum*	14.47 ± 0.46	1.80 × 10^7^	24.99^∗^ ± 3.12	7.68 ± 6.09	0.12 ± 0.05
*A. mytili*	14.34 ± 0.03	2.57 × 10^7^	56.98 ± 14.71	19.5 ± 15.6	0.63 ± 1.08
*A. nitrofigilis*	17.99 ± 0.21	3.52 × 10^6^	58.06 ± 2.57	11.3 ± 0.96	0.03 ± 0.03
*A. skirrowii*	13.74 ± 0.21	1.12 × 10^7^	50.32 ± 4.77	9.38 ± 1.85	ND
*A. suis*	14.78 ± 0.30	5.77 × 10^6^	71.31^∗^ ± 4.2	19.09 ± 2.59	0.01 ± 0
*A. thereius*	15.54 ± 0.33	8.69 × 10^6^	55.65 ± 13.2	9.04 ± 2.88	ND
*A. trophiarum*	14.94 ± 0.61	7.07 × 10^7^	39.4 ± 23.57	6.96 ± 6.15	0.13 ± 0.21
*A. venerupis*	14.77 ± 0.22	2.06 × 10^7^	30.28 ± 14.21	8.44 ± 4.88	ND
Average	14.36 ± 1.20	2.59 × 10^7^	53.31 ± 21.97	12.32 ± 7.85	0.09 ± 0.27

*^*a*^Percentage of copy numbers detected in relation to the total copy number obtained from the bacterial model of 100% live cells. ^∗^Mean percentage was statistically different from the rest (*P* = 0.05). LC, live cells; DC, dead cells; ND, no detection.*

### Quantification of *Arcobacter* spp. in Seafood

Mussel and oyster samples were artificially inoculated with *A. butzleri* live and dead cells, in order to assess the interference from the shellfish tissues to the reactivity of the PMA. Results showed that, in the first place, there was no background *Arcobacter* DNA in the blank mussel and oyster samples; in the second place, when the cells were dead, the resulting Ct values suggest that DNA may have been lost or degraded during the extraction process (Supplementary Figure S3). However, the use of PMA improved the reduction of the signal from DNA of dead cells achieving the corresponding percentages of copies.

**FIGURE 3:**
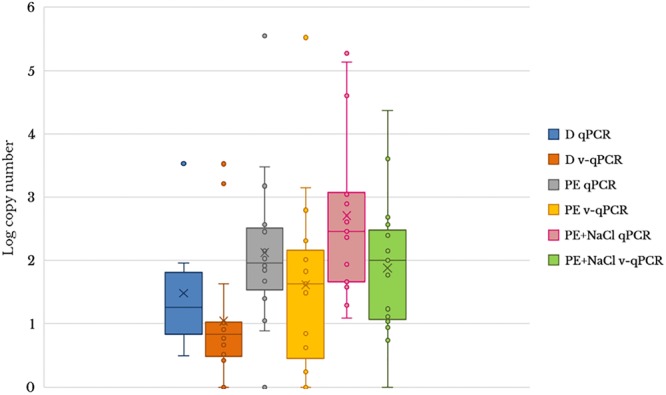
Box plot comparing 23S q-PCR and 23S v-qPCR results for detection of *Arcobacter* spp. from 17 shellfish samples. D, direct samples; PE, post-enrichment in Arcobacter-CAT broth; PE + NaCl, post-enrichment in Arcobacter-CAT broth supplemented with 2.5% NaCl.

To evaluate the usefulness of the v-qPCR method, a total of 17 different raw shellfish samples were tested for the presence of viable *Arcobacter* spp. cells and the results were compared with those obtained from two different enrichment broths in addition to direct culture isolation (Figure 3). Only 5.9% (1/17) of the samples were positive by direct plating on BA. Culture after pre-enrichment in blood agar and marine agar yielded a 29.4 and 35.3% of positive samples, respectively. However, the number of positive samples increased when the v-qPCR was used. As expected, the number of copies/g of raw shellfish samples were lower than those from pre-enriched samples (PE). Differences in the number of copies were observed between samples treated or not treated with PMA, with a reduction in the copy number seen for those treated with PMA (Figure 3).

The effect of PMA on the reduction of qPCR signal from shellfish samples showed that the use of PMA reduced the signal and resulted in a lower copy number being detected (Table 3). The reduction effect (ΔCt) from direct samples and those pre-enriched in Arcobacter-CAT broth were similar ΔCt _Dsamples_ = 1.05 and ΔCt _PEsamples_ = 1.04. However, a higher reduction is observed in the samples pre-enriched in Arcobacter-CAT broth supplemented with 2.5% NaCl ΔCt_PE+NaCl samples_ = 3.27.

**Table 3:** Quantitative detection of 23S copy numbers of *Arcobacter* spp. in 17 seafoods samples by qPCR and v-qPCR and comparison of results obtained by culture isolation.

	Log number of copies/g of flesh and intervalval liquid of shellfish						Culture			
					PE + NaCl	PE + NaCl
Sample	D qPCR	D v-qPCR	PE qPCR	PE v-qPCR	qPCR	v-qPCR	D-BA^a^	D-MA^b^	PE-BA^c^	PE-MA^d^
Mussel 1	4.23	ND	5.20	5.19	5.63	5.36	–	–	–	–
Mussel 2	4.72	4.13	4.22	ND	8.45	6.78	–	–	–	–
Mussel 3	4.43	3.97	6.65	3.45	4.26	4.28	–	–	–	–
Mussel 4	4.61	3.84	4.06	3.41	8.31	7.55	–	–	–	–
Mussel 5	5.14	3.69	5.03	4.02	4.75	3.91	–	–	–	–
Oyster 1	3.67	3.60	5.34	5.01	5.55	5.18	–	–	–	–
Oyster 2	4.00	3.95	5.63	5.48	5.11	4.94	–	–	–	–
Razor clam 1	4.84	4.21	ND	ND	5.79	ND	–	–	–	–
Razor clam 2	4.32	3.63	4.58	4.81	4.84	4.41	–	–	–	+
Razor clam 3	3.70	ND	5.74	5.97	6.27	5.57	–	–	+	–
Wedge clam 1	6.71	6.70	6.35	6.32	5.54	5.32	+	–	–	+
Wedge clam 2	6.73	6.39	8.73	8.70	6.07	5.86	–	–	+	–
Wedge clam 3	4.91	4.81	5.09	3.80	7.78	5.74	–	–	+	+
Wedge clam 4	3.92	4.13	4.85	4.66	4.46	4.34	–	–	–	–
Wedge clam 5	5.06	4.01	5.13	5.00	4.84	4.21	–	–	+	+
Wedge clam 6	4.13	4.19	5.32	4.08	6.22	4.12	–	–	–	+
Wedge clam 7	4.01	4.09	5.02	5.19	6.13	5.23	–	–	+	+
Average	4.65	3.84	5.11	4.42	5.88	4.87				

*D, direct samples; PE, post-enrichment in Arcobacter-CAT broth; PE + NaCl, post-enrichment in Arcobacter-CAT broth supplemented with 2.5% NaCl. ^*a*^D-BA, homogenized samples directly cultured onto blood agar and incubated at 30°C for 48 h. ^*b*^D-MA, homogenized samples directly cultured onto marine agar and incubated at 30°C for 48 h. ^*c*^PE-BA, post-enrichment in Arcobacter-CAT broth cultured onto blood agar and incubated at 30°C for 48 h. ^*d*^PE-MA, post-enrichment in Arcobacter-CAT broth supplemented with 2.5% NaCl cultured onto marine agar and incubated at 30°C for 48 h.*

## Discussion

The genus *Arcobacter* comprises species that have been considered zoonotic agents and emergent pathogens by the International Commission on Microbiological Specifications for Foods (ICMSF, 2002) i.e., *A. butzleri*. *Arcobacter* species have been recovered from a wide range of different food animals and food products worldwide (Collado and Figueras, 2011; Ferreira et al., 2016, 2017), among which seafood represents a reservoir of known and unknown *Arcobacter* species (Salas-Massó et al., 2016, 2018), posing a risk for the consumer. The detection of foodborne pathogens in shellfish is mainly based on culture techniques, which are time consuming and cannot detect VBNC cells; but also, in PCR, which cannot differentiate between DNA from live or dead cells, that could lead to unnecessary product recalls (Barbau-Piednoir et al., 2014; Gensberger et al., 2014; Zhang et al., 2016). Thus, for the first time in our study, the use of a viable-qPCR method based on specific primers for the detection of viable *Arcobacter* spp. cells in shellfish was developed.

Recently, Ferreira et al. (2017) reviewed the different molecular methods for the detection of *Arcobacter* spp.; most of them being multiplex PCR targeting only three species, *A. butzleri, A. cryaerophilus*, and *A. skirrowii* [i.e., m-PCR by Harmon and Wesley (1997); m-PCR by Houf et al. (2000); m-PCR by Kabeya et al. (2003); PCR by González et al. (2014)]. So far, four quantitative PCR (qPCR) have already been developed for *Arcobacter*. De Boer et al. (2013) and Webb et al. (2016) designed a qPCR specific for *A. butzleri* targeting the genes *hsp60* (encoding for a heat shock protein) and the *qhnDH* (encoding for the gamma subunit of quinohemoprotein amine dehydrogenase). However, Hausdorf et al. (2013) developed two q-PCR assays targeting both, the 16S and the 23S rRNA genes. The 16S q-PCR showed a reduced efficiency detecting *A. halophilus* and *A. marinus*, both species being common in shellfish (Salas-Massó et al., 2016). Thus, the 23S q-PCR was chosen to be coupled with PMA because of its higher efficiency detecting *Arcobacter* spp., and because the amplification product (233 bp) had an appropriate length for PMA experiments as previously reviewed by Fittipaldi et al. (2012). Although the authors tested the specificity of their primers with other genera different from *Arcobacter*, they did not test other bacteria like *Campylobacter*, which is closely related to *Arcobacter*, nor *Salmonella*, which is also commonly found in different types of food, and only 15 *Arcobacter* species were tested. After a modification in the forward primer we observed that no amplification from other species different from *Arcobacter* were obtained and all the 20 *Arcobacter* species tested were detected with no signal from the other genera tested.

One of the main problems regarding v-qPCR is the comparison of results among the different studies available in the literature, mainly due to the different conditions used in the experiments. A clear example of this is the study of Josefsen et al. (2010), they developed a PMA-qPCR targeting *Campylobacter* spp. in broiler carcasses, obtaining good results in the discrimination of dead cells even at bacterial concentration of 10^6^ CFU ⋅ mL^-1^. However, Pacholewicz et al. (2013) using the same PMA-qPCR protocol of Josefsen’s, did not obtain complete inhibition of the signal from dead *Campylobacter* cells at concentrations higher than 10^4^ CFU ⋅ mL^-1^. Similar results were obtained by Seinige et al. (2014) although they used a different qPCR protocol. Recently, Castro et al. (2018) evaluated the presence of *Campylobacter* cells in frozen and chilled broiler carcasses by means of a v-qPCR and they found a good discrimination between the different stages of the bacterial cells. Among other considerations, the differences between these studies could be attributed to the fact that none of the four studies mentioned above had the same PMA treatment. The light source varied in power and type (halogen lamps vs. LED devices); the PMA final concentration was different (i.e., 10, 20, 25.55, and 50 μg ⋅ mL^-1^); there were also variations in the incubation time and temperature, as well as the photoactivation time (1, 3, and 15 min). This is why the optimization and standardization of v-qPCR protocols are necessary. Additionally, the storage of a sample should also be taken into account, when comparing results, because as long as the storage step affects the viability of the cells, it would also influence the results obtained by the v-qPCR. In the study of Castro et al. (2018), they were led to the conclusion that *Campylobacter* spp. remain viable more frequently in chilled carcasses than in frozen ones. Further, Fernández-Piquer et al. (2012) showed that the number of *Arcobacter* cells are affected by the storage temperature of oysters. The number of *Arcobacter* cells increase after storage in comparison to the pre-storage oysters (Fernández-Piquer et al., 2012). Similar results were obtained for *Arcobacter buztleri* in broiler carcasses, indicating that this species can multiply during storage (Badilla-Ramírez et al., 2016).

Optimization of the use of DNA-intercalating dyes should be performed for each species due to the different sensitivity and integrity of the membrane and to diverse susceptibilities of different species to stress (Fittipaldi et al., 2012). Usually, the optimization of the protocol should include selection of dye (EMA or PMA), type of light used, time of photoactivation, concentration of the dye and of the bacteria, among other factors. In this work PMA was chosen as it has been demonstrated that PMA has a lower cytotoxicity than EMA (Fittipaldi et al., 2012 and references therein). Hrušková et al. (2013) performed a study where they evaluated the proportion of viable and dead cells of *A. butzleri* and *A. cryaerophilus* in biofilms. In that study they were the first researchers to test this genus using both EMA and PMA. Blockage of amplification of DNA from dead *A. butzleri* cells was achieved using EMA 25 μg ⋅ mL^-1^ or PMA 0.2 μmol ⋅ L^-1^. However, *A. cryaerophilus* was more sensitive to both dyes, i.e., EMA 1 μg ⋅ mL^-1^ or PMA 0.02 μmol ⋅ L^-1^. Recently, Webb et al. (2016) described a q-PCR for which they used an EMA final concentration of 100 μg ⋅ mL^-1^ as a pre-treatment for detecting *A. butzleri* cells in wastewater samples. Nevertheless, this protocol was originally designed for *Campylobacter jejuni*, and according to the results from Hrušková et al. (2013), this concentration would block amplification of DNA from viable cells leading to an underestimation of the number of live cells detected by their q-PCR. However, in our study we found that PMA at a concentration of 20 μmol ⋅ L^-1^ was optimal. According to Hrušková et al. (2013) this concentration would not block the signal from *A. cryaerophilus* cells, but it would from those of *A. butzleri*. On the contrary, we did not observe any reduction in the signal from live *A. butzleri* cells at 20 μmol ⋅ L^-1^. One of the possible reasons for this discrepancy could be the photoactivation source. In both works (Hrušková et al., 2013; Webb et al., 2016) they used a halogen lamp, which has shown to heat the samples leading to a possible higher susceptibility of the cells to the dyes. The use of devices like the one used in this study (Phast Blue which uses LED, thus generating only negligible heat) would make the use of viable dyes more standardized (Fittipaldi et al., 2012).

When the PMA-based v-qPCR was used for testing model mixtures of cells (live and/or dead), the percentages obtained for the different ratios of live and dead cells was in general terms good, which are in concordance with the observations reported by Hrušková et al. (2013) for *A. butzleri* and *A. cryaerophilus*, where the PMA method was good enough to discern viable cells from dead cells. These experiments with model mixtures have demonstrated the ability for differentiation of cell status by PMA in other bacterial species such as *Listeria innocua* (Løvdal et al., 2011; Soejima et al., 2011), and *Enterobacter sakazakii* (Cawthorn and Witthuhn, 2008). However, in the present work, when PMA was applied to the model 50% LC + 50% DC for *A. suis*, an overestimation of the proportion of live cells occurred, indicating that PMA at that ratio was not capable of binding to all the DNA present in dead or damaged cells. On the other hand, *A. ellisii* and *A. molluscorum* showed an underestimation in the percentage of live cells in the 50:50 model. Fittipaldi et al. (2012) in their review, showed that different proportions of dead cells in a sample can determine an increase/decrease in the Ct value of the samples treated with PMA. In this case for the species *A. suis, A. ellisii*, and *A. molluscorum* the ratio 50:50 altered the effectiveness of the method. Nevertheless, it should be taken into account that the v-qPCR has been designed for detecting the genus *Arcobacter* and not specific species. As a tool for food safety, on average the PMA method presented good results that should aid in preventing unnecessary, costly food recalls.

To date, only two studies have investigated the effectivity of a v-qPCR methodology in seafood samples. Quijada et al. (2016) showed that PMA activity was not affected by the processing of such a complex matrix as are clams. However, Zhu et al. (2012) when analyzing different seafood (including oysters, scallops and crabs) observed that samples with turbidities greater than 10 Nephelometric Turbidity Units (NTU) did not adequately inhibit the amplification of DNA from dead cells. Our results showed that, when processing *A. butzleri* spiked shellfish samples, the PMA method was effective for inhibiting the signal from the dead cells, in concordance with the results obtained by Quijada et al. (2016). However, when PMA was not used, there was also a reduction in the number of copies detected from the model mixtures of live and dead cells, indicating that part of the dead cells and free DNA added to the spiked sample could have been lost during DNA extraction. It has been demonstrated that different DNA extraction protocols yield different quantity and/or quality of nucleic acid (Demeke et al., 2009 and references therein). Thus, DNA extraction may be an additional factor to include in future standardization of v-qPCR protocols. The v-qPCR method presented in this study, and tested in four different types of shellfish, could be used for studying *Arcobacter* in other different matrices, providing that the DNA extraction method is demonstrated to be sufficiently effective for the other matrix, as Quijada et al. (2016) have done for their enteric RNA and DNA viruses PMA protocol, that was applied in clams and “chorizo” sausages. Nevertheless, Fittipaldi et al. (2012) indicated in their review that EMA and PMA effectiveness is matrix dependent; this is why optimization of the protocols are highly recommended.

Some studies have used in parallel direct plating, post-enrichment culture and a direct multiplex-PCR for analyzing diverse types of samples (González et al., 2007; Collado and Figueras, 2011; Levican et al., 2016). In these studies, it was found that direct multiplex-PCR yielded the same or higher number of positive samples as culturing, with the exception of Levican et al. (2016) that reported just the opposite. In our study, we used specific qPCR and v-qPCR as tools for detection of *Arcobacter* in shellfish samples in parallel to four culturing approaches. As recommended by Salas-Massó et al. (2016), when analyzing seafood samples, in addition to the conventional approach as described in Levican et al. (2016), we included direct plating in marine agar and post-enrichment in Arcobacter-CAT broth + 2.5% NaCl and subculturing in marine agar. Salas-Massó et al. (2016) demonstrated that the use of this protocol in marine samples yielded c.a. 40% more positive culture samples for *Arcobacter* than when only the conventional approach was used for analyzing these samples. Through the use of culture-independent approaches (molecular biology), we obtained a higher number of positive samples than from the culture-based approaches. The presence of potential new unculturable *Arcobacter* species in marine samples have been demonstrated in several studies (Miller et al., 2007; Wesley and Miller, 2010; Collado and Figueras, 2011; Fernández-Piquer et al., 2012; King et al., 2012) that could favor the utilization of molecular tools over culture-based methodologies. While DNA-based approaches can detect cells in the VBNC state, a disadvantage is that free DNA is also detected from dead cells. Our results have shown that implementation of v-qPCR, using PMA, reduces the signal obtained from samples containing dead cells as compared to those analyzed by standard qPCR, indicating that amplification of free DNA, or that of dead cells, is being blocked as occurred for other bacterial species in many different samples (Kobayashi et al., 2009; Josefsen et al., 2010; Li and Chen, 2012; Zhu et al., 2012; Zhang et al., 2015). However, a greater reduction was observed in samples that corresponded to post-enrichment in Arcobacter-CAT broth + 2.5% NaCl. This phenomenon has been reported by Shi et al. (2011) where heat-killed cells were previously exposed to different concentrations of NaCl (0.125–10%), and they observed that the higher the osmotic shock, the greater is the signal reduction. This may be attributed to an osmotic destabilization of the cell membrane leading to more efficient dye uptake (Fittipaldi et al., 2012).

## Conclusion

This is the first report on the development of a viable q-PCR for selectively amplifying DNA from viable *Arcobacter* spp. cells in shellfish samples. The PMA protocol was optimized for 20 species of the genus *Arcobacter* taking into account diverse factors like the concentration of PMA, incubation time and temperature, photo-activation time or cell concentration. The usefulness of PMA was then extrapolated to a v-qPCR where different mixed ratios of viable and dead cells were used, obtaining satisfactory inhibition of DNA amplification from the different proportions of dead cells in 85% of the *Arcobacter* species tested. The demonstrated efficiency of the PMA v-qPCR was applied to real seafood matrices such as raw oysters and mussels. A general decrease in the number of copies was detected in spiked samples treated with and without PMA, probably associated to DNA extraction procedures for shellfish samples. However, when PMA was applied, a significant reduction in the signal of *Arcobacter* DNA was observed and this reduction increased when the DNA was extracted from post-enrichment broth containing 2.5% NaCl, favoring the penetration of the PMA into damaged cells.

With this study, we encourage the use and standardization of viable qPCR for rapid, specific detection of viable microorganisms of public health concern in food products. Thus, this work, if applied to *Arcobacter* species along with other hazardous bacteria and viruses, could contribute to improve the database for food safety authorities, when regulating for food safety and risk analysis regarding shellfish consumption. Moreover, it opens a new way to better study the potential role of *Arcobacter*, not only in estuarine and marine environments, where its associations with shellfish could have other unexplored roles, but also in other food matrices or environments like sewage where *Arcobacter* spp. are frequently recovered, and even for studies focused in the clinical aspects of these microorganisms.

## Note

A paper entitled “Revisiting the Taxonomy of the Genus *Arcobacter*: Getting Order From the Chaos” by Pérez-Cataluña et al. (2018) was published in Front. Microbiol. 2018 Sep 4; 9:2077. doi: 10.3389/fmicb.2018.02077, followed by an Erratum, while our manuscript was under review. Pérez-Cataluña et al. (2018) proposed the reassignment of several *Arcobacter* spp. to other genera including newly proposed taxa; however, we have retained presently valid nomenclature because the proposed names have not yet been validated.

## Data Availability

All datasets generated for this study are included in the manuscript and/or the Supplementary Files.

## Author Contributions

DDB, AW, KA, MF, and MDF designed the work. NS-M, QL, and WC performed the optimization protocol for PMA. NS-M carried out the setup of the viable q-PCR and tested shellfish samples. NS-M, DDB, KA, MF, and MDF wrote the manuscript.

## Conflict of Interest Statement

The authors declare that the research was conducted in the absence of any commercial or financial relationships that could be construed as a potential conflict of interest.

## References

[B1] Badilla-Ramírez Y. Fallas-Padilla K. L. Fernández-Jaramillo H. Arias-Echandi M. L. (2016). Survival capacity of *Arcobacter butzleri* inoculated in poultry meat at two different refrigeration temperatures. *Rev. Inst. Med. Trop. São Paulo* 58:22. 10.1590/S1678-9946201658022 27007565 PMC4804559

[B2] Barbau-Piednoir E. Mahillon J. Pillyser J. Coucke W. Roosens N. H. Botteldoorn N. (2014). Evaluation of viability-qPCR detection system on viable and dead *Salmonella* serovar Enteritidis. *J. Microbiol. Methods* 103 131–137. 10.1016/j.mimet.2014.06.003 24927988

[B3] Castro A. G. Dorneles E. M. Santos E. L. Alves T. M. Silva G. R. Figueiredo T. C. (2018). Viability of *Campylobacter* spp. in frozen and chilled broiler carcasses according to real-time PCR with propidium monoazide pretreatment. *Poult. Sci.* 97 1706–1711. 10.3382/ps/pey020 29471351

[B4] Cawthorn D. M. Witthuhn R. C. (2008). Selective PCR detection of viable *Enterobacter sakazakii* cells utilizing propidium monoazide or ethidium bromide monoazide. *J. Appl. Microbiol.* 105 1178–1185. 10.1111/j.1365-2672.2008.03851.x 18624747

[B5] Collado L. Figueras M. J. (2011). Taxonomy, epidemiology, and clinical relevance of the genus *Arcobacter*. *Clin. Microbiol. Rev.* 24 174–192. 10.1128/CMR.00034-10 21233511 PMC3021208

[B6] Collado L. Guarro J. Figueras M. J. (2009). Prevalence of *Arcobacter* in meat and shellfish. *J. Food Protect.* 72 1102–1106. 10.4315/0362-028X-72.5.1102 19517742

[B7] De Boer R. F. Ott A. Güren P. Van Zanten E. Van Belkum A. Kooistra-Smid A. M. D. (2013). Detection of campylobacter species and *Arcobacter butzleri* in stool samples by use of real-time multiplex PCR. *J. Clin. Microbiol.* 51 253–259. 10.1128/JCM.01716-12 23152553 PMC3536235

[B8] Demeke T. Ratnayaka I. Phan A. (2009). Effects of DNA extraction and purification methods on real-time quantitative PCR analysis of roundup ready soybean. *J. AOAC Int.* 92 1136–1144. 19714982

[B9] EFSA and ECDC (2016). The European Union summary report on trends and sources of zoonoses, zoonotic agents and food-borne outbreaks in 2015. *EFSA J.* 14:4634. 32625371 10.2903/j.efsa.2017.5077 PMC7009962

[B10] Elizaquível P. Aznar R. Sánchez G. (2014). Recent developments in the use of viability dyes and quantitative PCR in the food microbiology field. *J. Appl. Microbiol.* 116 1–13. 10.1111/jam.12365 24119073

[B11] Fernández-Piquer J. Bowman J. Ross T. Tamplin M. (2012). Molecular analysis of the bacterial communities in the live Pacific oyster (*Crassostrea gigas*) and the influence of postharvest temperature on its structure. *J. Appl. Microbiol.* 112 1134–1143. 10.1111/j.1365-2672.2012.05287 22429335

[B12] Ferreira S. Oleastro M. Domingues F. C. (2017). “*Arcobacter* spp. in food chain—from culture to omics,” in *Food Borne Pathogens and Antibiotic Resistance* ed. Singh O. V. (Hoboken, NJ: Wiley-Blackwell) 73–118. 10.1002/9781119139188

[B13] Ferreira S. Queiroz J. A. Oleastro M. Domingues F. C. (2016). Insights in the pathogenesis and resistance of *Arcobacter*: a review. *Crit. Rev. Microbiol.* 42 364–383. 10.3109/1040841X.2014.954523 25806423

[B14] Fittipaldi M. Nocker A. Codony F. (2012). Progress in understanding preferential detection of live cells using viability dyes in combination with DNA amplification. *J. Microbiol. Methods* 91 276–289. 10.1016/j.mimet.2012.08.007 22940102

[B15] Gensberger E. T. Polt M. Konrad-Köszler M. Kinner P. Sessitsch A. Kostić T. (2014). Evaluation of quantitative PCR combined with PMA treatment for molecular assessment of microbial water quality. *Water Res.* 15 367–376. 10.1016/j.watres.2014.09.022 25459225

[B16] González A. Botella S. Montes R. M. Moreno Y. Ferrus M. A. (2007). Direct detection and identification of *Arcobacter* species by multiplex PCR in chicken and wastewater samples from Spain. *J. Food Prot.* 70 341–347. 10.4315/0362-028X-70.2.341 17340867

[B17] González I. Fernández-Tomé S. García T. Martín R. (2014). Genus-specific PCR assay for screening *Arcobacter* spp. in chicken meat. *J. Sci. Food Agric.* 94 1218–1224. 10.1002/jsfa.6401 24105785

[B18] Harmon K. M. Wesley I. V. (1997). Multiplex PCR for the identification of *Arcobacter* and differentiation of *Arcobacter butzleri* from other *Arcobacters*. *Vet. Microbiol.* 58 215–227. 10.1016/S0378-1135(97)00151-X 9453132

[B19] Hausdorf L. Neumann M. Bergmann I. Sobiella K. Mundt K. Fröhling A. (2013). Occurrence and genetic diversity of *Arcobacter* spp. in a spinach-processing plant and evaluation of two Arcobacter-specific quantitative PCR assays. *Syst. Appl. Microbiol.* 36 235–243. 10.1016/j.syapm.2013.02.003 23561260

[B20] Houf K. Tutenel A. De Zutter L. Van Hoof J. Vandamme P. (2000). Development of a multiplex PCR assay for the simultaneous detection and identification of *Arcobacter butzleri, Arcobacter cryaerophilus* and *Arcobacter skirrowii*. *FEMS Microbiol. Lett.* 193 89–94. 10.1111/j.1574-6968.2000.tb09407.x 11094284

[B21] Hrušková L. Mot’ková P. Vytřasová J. (2013). Multiplex polymerase chain reaction using ethidium monoazide and propidium monoazide for distinguishing viable and dead cells of *Arcobacters* in biofilm. *Can. J. Microbiol.* 59 797–802. 10.1139/cjm-2013-0635 24313452

[B22] Hsu T. T. D. Lee J. (2015). Global distribution and prevalence of *Arcobacter* in food and water. *Zoonoses Public Health* 62 579–589. 10.1111/zph.12215 26172312

[B23] ICMSF (2002). “ICMSF,” in *Microbiological Testing in Food Safety Management* ed. Tompkin R. B. (New York, NY: Kluwer Academic) 171.

[B24] Josefsen M. H. Löfström C. Hansen T. B. Christensen L. S. Olsen J. E. Hoorfar J. (2010). Rapid quantification of viable *Campylobacter* bacteria on chicken carcasses, using real-time PCR and propidium monoazide treatment, as a tool for quantitative risk assessment. *Appl. Environ. Microbiol.* 76 5097–5104. 10.1128/AEM.00411-10 20562292 PMC2916463

[B25] Kabeya H. Kobayashi Y. Maruyama S. Mikami T. (2003). One-step polymerase chain reaction-based typing of *Arcobacter* species. *Int. J. Food Microbiol.* 81 163–168. 10.1016/S0168-1605(02)00197-6 12457591

[B26] King G. Judd C. Kuske C. Smith C. (2012). Analysis of stomach and gut microbiomes of the eastern oyster (*Crassostrea virginica*) from coastal Louisiana. USA. *PLoS One* 7:e51475. 10.1371/journal.pone.0051475 23251548 PMC3520802

[B27] Kobayashi H. Oethinger M. Tuohy M. J. Hall G. S. Bauer T. W. (2009). Improving clinical significance of PCR: use of propidium monoazide to distinguish viable from dead *Staphylococcus aureus* and *Staphylococcus epidermidis*. *J. Orthop. Res.* 27 1243–1247. 10.1002/jor.20872 19322790

[B28] Leoni F. Chierichetti S. Santarelli S. Talevi G. Masini L. Bartolini C. (2017). Occurrence of *Arcobacter* spp. and correlation with the bacterial indicator of faecal contamination *Escherichia coli* in bivalve molluscs from the Central Adriatic, Italy. *Int. J. Food Microbiol.* 245 6–12. 10.1016/j.ijfoodmicro.2017.01.006 28113092

[B29] Levican A. Collado L. Figueras M. J. (2016). The use of two culturing methods in parallel reveals a high prevalence and diversity of *Arcobacter* spp. in a wastewater treatment plant. *Biomed. Res.* 2016:8132058. 10.1155/2016/8132058 27981053 PMC5131228

[B30] Li B. Chen J. Q. (2012). Real-time PCR methodology for selective detection of viable *Escherichia coli* O157:H7 cells by targeting Z3276 as a genetic marker. *Appl. Environ. Microbiol.* 78 5297–5304. 10.1128/AEM.00794-12 22635992 PMC3416439

[B31] Løvdal T. Hovda M. B. Björkblom B. Møller S. G. (2011). Propidium monoazide combined with real-time quantitative PCR underestimates heat-killed *Listeria innocua*. *J. Microbiol. Methods* 85 164–169. 10.1016/j.mimet.2011.01.027 21324348

[B32] Miller W. G. Parker C. T. Rubenfield M. Mendz G. L. Wösten M. M. Ussery D. W. (2007). The complete genome sequence and analysis of the epsilon proteobacterium *Arcobacter butzleri*. *PLoS One* 26 2:e1358. 10.1371/journal.pone.0001358 18159241 PMC2147049

[B33] Nieva-Echevarria B. Martinez-Malaxetxebarria I. Girbau C. Alonso R. Fernández Astorga A. (2013). Prevalence and genetic diversity of *Arcobacter* in food products in the north of Spain. *J. Food Prot.* 76 1447–1450. 10.4315/0362-028X.JFP-13-014 23905804

[B34] Nocker A. Cheung C. Y. Camper A. K. (2006). Comparison of propidium monoazide with ethidium monoazide for differentiation of live vs. dead bacteria by selective removal of DNA from dead cells. *J. Microbiol. Methods* 67 310–320. 10.1016/j.mimet.2006.04.015 16753236

[B35] Nocker A. Mazza A. Masson L. Camper A. K. Brousseau R. (2009). Selective detection of live bacteria combining propidium monoazide sample treatment with microarray technology. *J. Microbiol. Methods* 76 253–261. 10.1016/j.mimet.2008.11.004 19103234

[B36] Pacholewicz E. Swart A. Lipman L. J. A. Wagenaar J. A. Havelaar A. H. Duim B. (2013). Propidium monoazide does not fully inhibit the detection of dead *Campylobacter* on broiler chicken carcasses by qPCR. *J. Microbiol. Methods* 95 32–38. 10.1016/j.mimet.2013.06.003 23811205

[B37] Pérez-Cataluña A. Salas-Massó N. Diéguez A. L. Balboa S. Lema A. Romalde J. L. (2018). Revisiting the taxonomy of the genus *Arcobacter*: getting order from the chaos. *Front. Microbiol.* 9:2077. 10.3389/fmicb.2018.02077 30233547 PMC6131481

[B38] Quijada N. M. Fongaro G. Barardi C. R. Hernández M. Rodríguez-Lázaro D. (2016). Propidium Monoazide integrated with qPCR enables the detection and enumeration of infectious enteric RNA and DNA viruses in clam and fermented sausages. *Front. Microbiol.* 15:2008. 10.3389/fmicb.2016.02008 28018329 PMC5156952

[B39] Reyneke B. Ndlovu T. Khan S. Khan W. (2017). Comparison of EMA-, PMA- and DNase qPCR for the determination of microbial cell viability. *Appl. Microbiol. Biotechnol.* 101 7371–7383. 10.1007/s00253-017-8471-6 28875372

[B40] Salas-Massó N. Andree K. B. Furones M. D. Figueras M. J. (2016). Enhanced recovery of *Arcobacter* spp. using NaCl in culture media and re-assessment of the traits of *Arcobacter marinus* and *Arcobacter halophilus* isolated from marine water and shellfish. *Sci. Total Environ.* 56 1355–1361. 10.1016/j.scitotenv.2016.05.197 27282494

[B41] Salas-Massó N. Figueras M. J. Andree K. B. Furones M. D. (2018). Do the *Escherichia coli* European Union shellfish safety standards predict the presence of *Arcobacter* spp., a potential zoonotic pathogen? *Sci. Total Environ.* 15 1171–1179. 10.1016/j.scitotenv.2017.12.178 29929229

[B42] Seinige D. Krischek C. Klein G. Kehrenberg C. (2014). Comparative analysis and limitations of ethidium monoazide and propidium monoazide treatments for the differentiation of viable and nonviable *Campylobacter* cells. *Appl. Environ. Microbiol.* 80 2186–2192. 10.1128/AEM.03962-13 24487529 PMC3993131

[B43] Shi H. Xu W. Luo Y. Chen L. Liang Z. Zhou X. (2011). The effect of various environmental factors on the ethidium monoazide and quantitative PCR method to detect viable bacteria. *J. Appl. Microbiol.* 111 1194–1204. 10.1111/j.1365-2672.2011.05125.x 21848696

[B44] Soejima T. Schlitt-Dittrich F. Yoshida S. (2011). Polymerase chain reaction amplification length-dependent ethidium monoazide suppression power for heat-killed cells of *Enterobacteriaceae*. *Anal. Biochem.* 418 37–43. 10.1016/j.ab.2011.06.027 21771573

[B45] Vandamme P. Falsen E. Rossau R. Hoste B. Segers P. Tytgat R. (1991). Revision of *Campylobacter, Helicobacter*, and *Wolinella* taxonomy: emendation of generic descriptions and proposal of *Arcobacter* gen. nov. *Int. J. Syst. Bacteriol.* 41 88–103. 10.1099/00207713-41-1-88 1704793

[B46] Webb A. L. Taboada E. N. Selinger L. B. Boras V. F. Inglis G. D. (2016). Efficacy of wastewater treatment on *Arcobacter butzleri* density and strain diversity. *Water Res.* 105 291–296. 10.1016/j.watres.2016.09.003 27636152

[B47] Wesley I. V. Miller G. W. (2010). “*Arcobacter*: an opportunistic human food-borne pathogen?,” in *Emerging Infections 9* eds Scheld W. M. Grayson M. L. Hughes J. M. (Washington, DC: ASM Press) 185–211. 10.12691/jaem-2-2-5

[B48] Zeng D. Chen Z. Jiang Y. Xue F. Li B. (2016). Advances and challenges in viability detection of foodborne pathogens. *Front. Microbiol.* 22:1833. 10.3389/fmicb.2016.01833 27920757 PMC5118415

[B49] Zhang H. N. Hou P. B. Chen Y. Z. Ma Y. Li X. P. Lv H. (2016). Prevalence of foodborne pathogens in cooked meat and seafood from 2010 to 2013 in Shandong province, China. *Iran J. Public Health* 45 1577–1585. 28053923 PMC5207098

[B50] Zhang Z. Liu W. Xu H. Aguilar Z. P. Shah N. P. Wei H. (2015). Propidium monoazide combined with real-time PCR for selective detection of viable *Staphylococcus aureus* in milk powder and meat products. *J. Dairy Sci.* 98 1625–1633. 10.3168/jds.2014-8938 25582587

[B51] Zhu R. G. Li T. P. Jia Y. F. Song L. F. (2012). Quantitative study of viable *Vibrio parahaemolyticus* cells in raw seafood using propidium monoazide in combination with quantitative PCR. *J. Microbiol. Methods* 90 262–266. 10.1016/j.mimet.2012.05.019 22677606

