# Supplementary material for: The Use of a DNA-Intercalating Dye for Quantitative Detection of Viable Arcobacter spp. Cells (v-qPCR) in Shellfish
Source: Front Microbiol. 2019 Feb 28;10:368. doi: 10.3389/fmicb.2019.00368 (PMC6403187; doi:10.3389/fmicb.2019.00368)
Supplement: Supplementary file 1 [file Table_1.DOCX]

**Suplementary material**

**Figure S1A**. Different concentration of PMA using 1ng/μl DNA. Lines 1 and 2: 20μM PMA; 3 and 4: 2μM PMA; 5 and 6: 0.2 μM PMA; 7 and 8: 0.02μM PMA; 9 and 10 no PMA; 11: negative control.


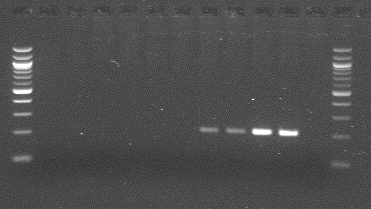


L 1 2 3 4 5 6 7 8 9 10 11 L

**Figure S1B.** Different concentration of *A. butzleri* DNA using 0.2 μM PMA. Lines1 and 2: 0 ng/μl DNA; 3 and 4: 1 ng/μl DNA; 5 and 6: 2 ng/μl DNA; 7 and 8: 10 ng/μl DNA; 9 and 10: 20 ng/μl; 11: 1 ng/μl of *A. butzleri* DNA but no PMA added; 12: negative control.

L 1 2 3 4 5 6 7 8 9 10 11 12 L


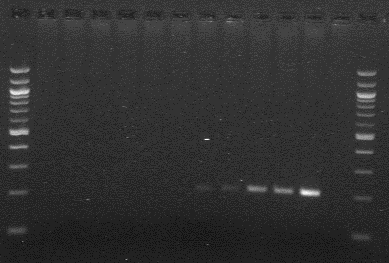


**Figure S1C.** Different concentration of PMA using different concentration of *A. butzleri* DNA. Lines 1 and 2: 10ng/μl DNA + 2μM PMA; 3 and 4: 20ng/μl DNA + 2μM PMA; 5 and 6: 10ng/μl DNA + 20μM PMA; 7 and 8: 20ng/μl DNA + 20μM PMA; 9: 1ng/μl DNA + no PMA; 11: negative control.


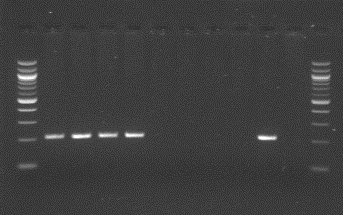


L 1 2 3 4 5 6 7 8 9 10 L

**Figure S1D.** Effect of time exposure to LED using 10ng/μl *A. butzleri* DNA and 0.2 μM PMA: 1 and 2: 7.5 min; 3 and 4: 15 min; 5 and 6: 30 min; 7 and 8: 60 min; 9 and 10: 60 min photoactivation without addition of PMA; 11: positive control 1 ng/μl *A. butzleri* DNA without PMA nor photoactivation; 12: negative control.


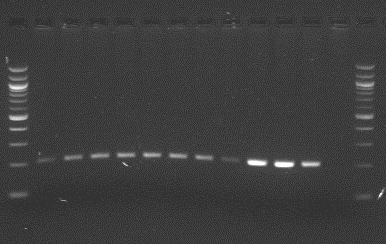


L 1 2 3 4 5 6 7 8 9 10 11 12 L

**Figure S1E**. Different concentration of PMA that were photoactivated with the following addition of 1ng/μl DNA. Lines 1: 20μM; 2: 2μM; 3: 0.2μM; 4: 0.02μM; 5: positive control 1ng/μl *A. butzleri* DNA no PMA; 6: negative control.


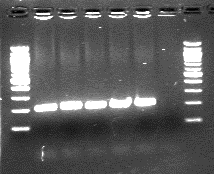


L 1 2 3 4 5 6 L

**Figure S2A.** Testing the efficiency of the PMA when working with DNA extracted from pure cultures of live (LC) or dead (DC) *A. butzleri* cells OD_550_=0.250 in a final volume of 25µl and treated or not with PMA. Line 1: LC no PMA added; 2: LC + 50µM PMA; 3: LC + 20µM PMA; 4: LC + 2µM PMA; 5: LC + 0.2µM PMA; 6: DC no PMA added; 7: DC + 50µM PMA; 8: DC + 20µM PMA; 9: DC + 2µM PMA;10: DC + 0.2µM PMA; 11: positive control 10ng/µl DNA *A. butzleri*; 12: negative control.

L 1 2 3 4 5 6 7 8 9 10 11 12 L


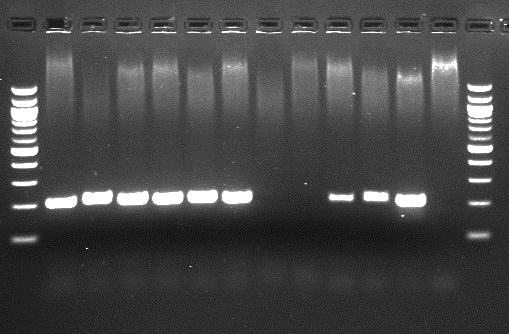


**Figure S2B.** Testing the efficiency of the PMA when working with DNA extracted from pure cultures of dead (DC) *A. butzleri* cells at different concentrations. Line 1: DC OD_550_=0.8; 2: DC OD_550_=0.4; 3: DC OD_550_=0.250; 4: DC OD_550_=0.8+ 20µM PMA; 5: DC OD_550_=0.4+ 20µM PMA; 6: DC OD_550_=0.250+ 20µM PMA; 7: positive control 10ng/µl DNA *A. butzleri*; 8: negative control.


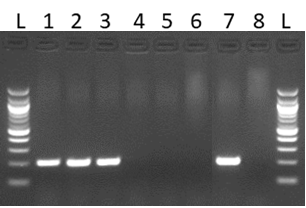


**Figure S3.** Percentage of detection of 23S *A. butzleri* copy numbers using qPCR and v-qPCR in mussels and oysters’ tissues using model mixtures of an initial inoculum (OD_550_=0.250) of live (LC) and dead (DC) cells, consisting in 100% LC; 50% LC + 50% DC and 100% DC.

**Table S1**. Species used for testing the specificity of the primers.

| Bacteria | Strain | Donor |
| --- | --- | --- |
| *Campylobacter coli* | CCUG 11283 | Provided by CCUG |
| *Campylobacter jejuni* | CCUG 11284 | Provided by CCUG |
| *Campylobacter lari* | CCUG 18267 | Provided by CCUG |
| *Campylobacter mucosilis* | CCUG 6822 | Provided by CCUG |
| *Campylobacter upsaliensis* | CCUG 14913 | Provided by CCUG |
| *Campylobacter sputorum ss.spo* | CCUG 9728 | Provided by CCUG |
| *Campylobacter fetus subsq. Fetus* | CCUG 6823A | Provided by CCUG |
| *Campylobacter concisus* | CCUG 131444 | Provided by CCUG |
| *Campylobacter hyointestinalis* | CCUG 19512 | Provided by CCUG |
| *Salmonella* Enteritidis | 92243/nybol 3L | Own strain |
| *Salmonella* Typhimurium | DVI-Jeo 3979 Jgt.110 | Own strain |
| *Enterococcus faecalis* | ATCC 29212 | Provided by ATCC |
| *Enterococcus faecium* | CCUG 47860 | Provided by CCUG |
| *Escherichia coli* | CCUG 17620 | Provided by CCUG |
| *Streptococcus pneunomiae* | ATCC 49619 | Provided by ATCC |
| *Proteus hauseri* | CCUG 36761 | Provided by CCUG |
| *Citrobacter freundii* | CCUG 418^T^ | Provided by CCUG |
| *Yersinia ruckeri* | ATCC 29473 | Provided by ATCC |
| *Arcobacter. aquimarinus* | CECT 8442^T^ | Own strain |
| *Arcobacter bivalviorum* | CECT 7836^T^ | Own strain |
| *Arcobacter butzleri* | LMG 10828^T^ | Provided by LMG |
| *Arcobacter cibarius* | CECT 7203^T^ | Provided by CECT |
| *Arcobacter cloacae* | CECT 7834^T^ | Own strain |
| *Arcobacter cryaerophilus* | LMG 9904^T^ | Provided by LMG |
| *Arcobacter defluvii* | CECT 7697^T^ | Own strain |
| *Arcobacter ebronensis* | CECT 8441^T^ | Own strain |
| *Arcobacter ellisii* | CECT 7837^T^ | Own strain |
| *Arcobacter halophilus* | LA31B^T^ | Provided by Dr Maqsudul Alam (University of Hawaii) |
| *Arcobacter lanthieri* | LMG 28516^T^ | Provided by LMG |
| *Arcobacter marinus* | CECT 7727^T^ | Provided by CECT |
| *Arcobacter molluscorum* | CECT 7696^T^ | Own strain |
| *Arcobacter mytili* | CECT 7386^T^ | Own strain |
| *Arcobacter nitrofigilis* | CECT 7204^T^ | Provided by CECT |
| *Arcobacter skirrowii* | LMG 6621^T^ | Provided by LMG |
| *Arcobacter suis* | CECT 7833^T^ | Own strain |
| *Arcobacter thereius* | LMG 24486^T^ | Provided by LMG |
| *Arcobacter trophiarum* | LMG 25534^T^ | Provided by LMG |
| *Arcobacter venerupis* | CECT 7836^T^ | Own strain |
